# An intact model for quantifying functional selectivity

**DOI:** 10.1038/s41598-019-39000-z

**Published:** 2019-02-22

**Authors:** Xiao Zhu, David B. Finlay, Michelle Glass, Stephen B. Duffull

**Affiliations:** 1 Otago Pharmacometrics Group, School of Pharmacy, University of Otago, Dunedin, New Zealand; 2 Department of Pharmacology and Clinical Pharmacology, Faculty of Medical and Health Sciences, University of Auckland, Auckland, New Zealand

## Abstract

A ligand that acts on a target receptor to activate particular multiple signalling pathways with activity that is distinct from other ligands is termed *ligand bias*. Quantification of ligand bias is based on applying the operational model to each pathway separately and subsequent calculation of the ligand bias metric (ΔΔ*logR*). This approach implies independence among different pathways and causes propagation of error in the calculation. Here, we propose a semi-mechanism-based model which allows for receptor selectivity across all the pathways simultaneously (termed the ‘intact operational model’). The power of the intact model for detecting ligand bias was evaluated via stochastic simulation estimation studies. It was also applied to two examples: (1) opposing effects of Gi/Gs signalling of α2-adrenergic receptors and (2) simultaneous measurement of arachidonic acid release and inositol phosphate accumulation following 5-HT_2C_ receptor activation. The intact operational model demonstrated greater power to detect ligand bias in the simulation. In the applications, it provided better precision of estimation and identified biased ligands that were missed by analysis of traditional methods. Issues identified in both examples might lead to different interpretations of the data. The intact operational model may elucidate greater understanding of the underlying mechanisms of functional selectivity.

## Introduction

Different ligands can differentially activate multiple signalling pathways when coupled to a single receptor. This feature is termed functional selectivity^1^. This complex pharmacological process involves multiple biological steps, such as receptor binding and activation of a range of intracellular signalling pathways. Simplified models have been adopted to delineate this phenomenon, in which each pathway is considered independently^2,3^.

In current practice, the most commonly used simplified model is the operational model, proposed in 1983 by Black and Leff^4^. In this approach, the operational model is applied separately to each pathway^5^. Since it is known that the pathways are linked biologically, this constitutes a simplification by assuming the pathways are independent. In modelling terms, this would be described as *marginal* and thus we denote this as ‘the marginal operational model’. When the operational model is applied in isolation, a composite parameter, *R* (the transduction coefficient; the ratio of the transducer ratio, *τ*, and functional affinity, *K*_*A*_), is obtained for quantifying the effect of a ligand on a single pathway. Ligand bias (the relative preference of a ligand for a particular pathway) is then computed in a *post hoc* analysis where a ligand’s transduction coefficient is normalised to that of a reference ligand in order to accommodate system and observational bias^6^. This yields the normalised transduction coefficient Δ*logR*. This value is then further normalised such that a ligand’s transduction coefficient for one pathway is related to its Δ*logR* for a second pathway. This second normalisation step provides a widely reported metric for ligand bias, ΔΔ*logR*. Calculating this metric for a given ligand in multiple pairwise pathway comparisons will formulate a signature profile of biases (preferences) across the range of pathways of interest, and these biases are often displayed as a radar plot^7^.

The use of the marginal operational model makes the assumption that each signalling pathway is independent. This precludes insights pertaining to functional selectivity from observed phenomena, such as the natural correlation of a ligand’s *C*_50_ values in different pathways. In addition, there is propagation of errors associated with the required *post hoc* parameter manipulation when the marginal operational model is used as the basis for determining ligand bias, due to the requirement for two successive normalisation steps.

In order to reflect the linkages between different signalling pathways, a mechanism-based three-state model has been introduced^8^. Within this model framework, equilibria are linked between different receptor conformational states and there is mutual depletion of these receptor states. This means that *C*_50_ values should be exactly the same for different signalling pathways. This strict constraint makes the three-state model less flexible and limits its applicability.

Here, we propose a simple extension of the operational model to allow its use across all pathways simultaneously and provide a direct estimate of ΔΔ*logR*. This model allows all pathways to be simultaneously modelled. Since we believe this provides mechanistic insight into what we consider to be an intact system, we denote this model as ‘the intact operational model’. This approach may shed light into the underlying mechanism of functional selectivity and act as the first step in the development of a full mechanistic model of functional selectivity. In addition, by allowing data from different pathways to inform the common components of the intact model, then the parameters of this model (*e.g*. *logR*) should be more precisely estimated.

The aims of this study are (1) to describe the intact operation model, (2) to evaluate its performance compared to the commonly utilised marginal operational model via theoretical assessment (power analysis) and practical applications (two literature examples) and (3) to demonstrate how a go/no-go workflow decision for ligand bias experiments varies based on the choice of operational model.

The main experimental components of this paper are divided into three parts to reflect progressive flow in our study. In the first part, the derivation of the intact operational model and general aspects of the data analyses are provided as they relate to both theoretical evaluation and practical applications. In the second part, the newly proposed method is theoretically evaluated via stochastic simulation estimation studies. In the third part, the findings from theoretical evaluation are further evaluated via practical applications in two examples. We have kept notation here the same as the convention. We note that *R* is conventionally used to represent two separate entities, the receptor *R* (shown in schematics and mass balance equations) and the transduction coefficient. To differentiate these components, when the receptor *R* is unbound to a ligand, A, we add the subscript ‘ub’ (*R*_*ub*_), and when referring to the total receptor numbers we use *R*_*t*_. The transduction coefficient is indexed (if required) by either a numeric value (if more than one ligand is present), or by the pathway signal (*e.g*. ‘i’ for inhibitory pathway and ‘s’ for stimulatory pathways).

## Part I

### Derivation of intact operational model

#### Marginal operational model

**Figure 1:**
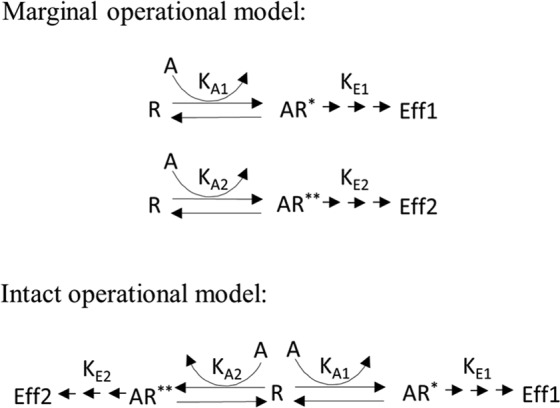
The schematic plot of the marginal operational model and the intact operational model.

The marginal operational model is illustrated in Fig. 1. In this representation, the different receptor conformational states are assumed to be independent. Within this model framework, the general operational model (Eq. 1, the same as Eq. 5 in van der Westhuizen *et al*., 2014) can be separately applied to each signalling pathway to fit the data.

1 $$E=Basal+\frac{({E}_{m}-Basal)}{1+{(\frac{(\frac{A}{{10}^{log{K}_{A}}}+1)}{{10}^{logR}\cdot A})}^{n}}$$

Here, *E*_*m*_ is the system maximal response, *Basal* is the baseline response in the absence of ligand and *n* is the Hill slope factor. These three parameters are system parameters. The ligand-specific parameters are *K*_*A*_ (the equilibrium dissociation constant), and *R* (the transduction coefficient, *τ*/*K*_*A*_). Conventionally, the parameters *K*_*A*_ and *R* are transformed into logarithms (*i.e*., ${10}^{log{K}_{A}}$ and 10^*logR*^).

By definition, the ligand bias metric is calculated as the difference between transduction coefficients from two pathways for the test ligand normalised to those of the reference ligand (Eq. 2):

2 $${\rm{\Delta }}{\rm{\Delta }}log{R}_{1-2}=(log{R}_{1}-log{R}_{1}^{REF})-(log{R}_{2}-log{R}_{2}^{REF})$$

Here, the superscript letter ‘REF’ indicates reference ligand. A subscript number “1” indicates pathway 1 and “2” indicates pathway 2.

The estimated standard error (SE) for test ligand is calculated using Eq. 3 (assuming independence – which for the standard *post hoc* analysis is reasonable).

3 $$S{E}_{({\rm{\Delta }}{\rm{\Delta }}log{R}_{1-2})}=\sqrt{S{E}_{(log{R}_{1})}^{2}+S{E}_{(log{R}_{1}^{REF})}^{2}+S{E}_{(log{R}_{2})}^{2}+S{E}_{(log{R}_{2}^{REF})}^{2}}$$

#### Intact operational model

The intact operational model is illustrated in Fig. 1. Here we see that equilibria are linked among different receptor conformational states and there is mutual depletion of these receptor states, though there may exist a theoretically unlimited number of active states. We only consider two active receptor conformations in the current study but this can be generalised to a greater number of states. The intact operational model (Eq. 4 for pathway 1 and Eq. 5 for pathway 2) is derived in detail in Appendix 1. Due to identifiability issues, it is not possible to estimate individual *K*_*A*_ values given only functional assay data. Hence, the information for those values is integrated into ${K}_{A}^{^{\prime} }$ (Eq. 6). Here, ${K}_{A}^{^{\prime} }$ is proportional to the harmonic mean of individual *K*_*A*_ values. Note that ${K}_{A}^{^{\prime} }$ is introduced to improve model identifiability but this should not prevent the researchers from estimating the individual *K*_*A*_ values when more information is at hand.

4 $$\frac{{E}_{1}}{{E}_{m1}}=\frac{1}{1+\frac{(\frac{A}{{K}_{A}^{^{\prime} }}+1)}{{R}_{1}\cdot A}}$$

5 $$\frac{{E}_{2}}{{E}_{m2}}=\frac{1}{1+\frac{(\frac{A}{{K}_{A}^{^{\prime} }}+1)}{{R}_{2}\cdot A}}$$

Here, ${K}_{A}^{^{\prime} }$ (Eq. 6) refers to the apparent equilibrium dissociation constant. *R*_1_ and *R*_2_ are the transduction coefficients (the ratio of transducer ratio and the equilibrium dissociation constant) for each of the two pathways.

6 $${K}_{A}^{^{\prime} }=\frac{1}{\frac{1}{{K}_{A1}}+\frac{1}{{K}_{A2}}}$$

The intact operational model can be generalised to account for non-zero basal (*Basal*), non-unity slope factor (*n*), and estimable maximal system response (*E*_*m*_), which we refer to as the general intact operational model (Eq. 7 for pathway 1 and Eq. 8 for pathway 2, detailed in Appendix 2). For the purpose of modelling, the parameters ${K}_{A}^{^{\prime} }$, *R*_1_ and *R*_2_ are transformed into logarithms (*i.e*., $\,{10}^{log{K}_{A}^{^{\prime} }}$, ${10}^{log{R}_{1}}$ and ${10}^{log{R}_{2}}$).

7 $${E}_{1}=Basa{l}_{1}+\frac{({E}_{m1}-Basa{l}_{1})}{1+{(\frac{(\frac{A}{{10}^{log{K}_{A}^{\text{'}}}}+1)}{{10}^{log{R}_{1}}\cdot A})}^{{n}_{1}}}$$

8 $${E}_{2}=Basa{l}_{2}+\frac{({E}_{m2}-Basa{l}_{2})}{1+{(\frac{(\frac{A}{{10}^{log{K}_{A}^{^{\prime} }}}+1)}{{10}^{log{R}_{2}}\cdot A})}^{{n}_{2}}}$$

9 $$\begin{array}{rcl}E & = & (Basa{l}_{1}+\frac{({E}_{m1}-Basa{l}_{1})}{1+{(\frac{(\frac{A}{{10}^{log{K}_{A}^{^{\prime} }}}+1)}{{10}^{log{R}_{1}}\cdot A})}^{{n}_{1}}}\,)\cdot {I}_{path=1}\\  &  & +(Basa{l}_{2}+\frac{({E}_{m2}-Basa{l}_{2})}{1+{(\frac{(\frac{A}{{10}^{log{K}_{A}^{^{\prime} }}}+1)}{{10}^{log{R}_{2}}\cdot A})}^{{n}_{2}}}\,)\cdot {I}_{path=2}\end{array}$$

In order to jointly model all the functional assay data, Eqs 7 and 8 are combined into Eq. 9. Here, *I*_*path*=1_ and *I*_*path*=2_ are indicator functions. *I*_*path*=1_ is equal to 1 for producing effect of pathway 1 and *I*_*path*=2_ is equal to 1 for producing effect of pathway 2.

In order to directly estimate ligand bias metric, ΔΔ*logR*_1−2_, a re-parameterisation is performed to explicitly incorporate ΔΔ*logR*_1−2_ as an estimated parameter into the model. From Eq. 2, *logR*_2_ is expressed as Eq. 10:

10 $$log{R}_{2}=log{R}_{1}-log{R}_{1}^{REF}+log{R}_{2}^{REF}-{\rm{\Delta }}{\rm{\Delta }}log{R}_{1-2}$$

Then, substituting Eq. 10 into Eq. 9 yields the intact operational model with ΔΔ*logR*_1−2_ as a directly estimated parameter (Eq. 11):

11 $$\begin{array}{rcl}E & = & (Basa{l}_{1}+\frac{({E}_{m1}-Basa{l}_{1})}{1+{(\frac{(\frac{A}{{10}^{log{K}_{A}^{\text{'}}}}+1)}{{10}^{log{R}_{1}}\cdot A})}^{{n}_{1}}}\,)\cdot {I}_{path=1}\\  &  & +(Basa{l}_{2}+\frac{({E}_{m2}-Basa{l}_{2})}{1+{(\frac{(\frac{A}{{10}^{log{K}_{A}^{\text{'}}}}+1)}{{10}^{(log{R}_{1}-log{R}_{1}^{REF}+log{R}_{2}^{REF}-{\rm{\Delta }}{\rm{\Delta }}log{R}_{1-2})}\cdot A})}^{{n}_{2}}}\,)\cdot {I}_{path=2}\end{array}$$

Thus, $log{K}_{A}^{^{\prime} }$, $log{R}_{1}^{REF}$, $log{R}_{2}^{REF}$, *logR*_1_ and ΔΔ*logR*_1−2_ are the ligand specific parameters that are incorporated directly into the intact operational model. Hence, the ligand bias metric (ΔΔ*logR*_1−2_) can be estimated and the estimated standard error can be obtained from modelling output.

### General aspects of data analysis

#### Model analysis

Parameter estimation was conducted using the software NONMEM 7.3.0 (ICON Development Solutions, Hanover, MD, USA)^9^. Model development was managed using Perl-Speaks-NONMEM 4.5.0, Pirana 2.9.0 and Xpose 4.5.3^10^. Due to the deterministic identifiability issue in the marginal operational model^11^, it was not possible to estimate *logK*_*A*_ values of full agonists from direct fitting to concentration-response curves. We followed the convention to arbitrarily set *logK*_*A*_ of the most efficacious ligand to 0 to circumvent this problem when it was necessary^11^. Model selection was informed by the minimum objective function value (MOFV)^12^. Model evaluation was based on the visual inspection of goodness of fit plots: (1) observed responses vs. predicted responses, (2) weighted residuals vs. predicted responses and (3) weighted residuals vs. ligand concentrations.

#### Test for ligand bias

Statistical analysis was performed using a Wald test on the ligand bias metric ΔΔ*logR*. Since the statistical significance of ligand bias for each test ligand was screened individually, the data from the reference ligand was used multiple times for constructing the ligand bias metrics ΔΔ*logR* for each ligand. To avoid the issue with multiple comparisons (inflation of type I error), the Bonferroni correction^13^ was used where the level of significance was set to *α*/*n*, where *α* was the desired overall significance level (*i.e*., 0.05 in this study) and *n* was the number of hypothesis tested.

## Part II

### Theoretical evaluation of the intact model

Since the intact operational model provides a more mechanism-based description of the biological system for functional selectivity, in current theoretical evaluation, we consider the intact operational model (Eq. 11) as the true model and then assess the influence of model misspecification (*i.e*., the marginal operational model) on the power of the ligand bias test via stochastic simulation estimation studies. In this circumstance we note that the intact operational model should not be inferior to the marginal operational model, rather, equivalent performance would favour the marginal operational model as a more parsimonious approach.

#### Power analysis process

The power analysis is divided into two steps. In the first step, we correct for false positive findings by calibrating the statistical test to achieve the appropriate type I error rate of 0.05. In the second step, the power was calculated for different levels of ligand bias with an appropriately calibrated type I error rate.

1. Calibration of the criterion for type I error (α = 0.05) via non-parametric approach
  Pseudo-experimental data were simulated from the true model (*i.e*., the intact operational model) under the null hypothesis (H_0_:ΔΔ*logR*_1−2_ = 0), based on the given parameter values and study design in Table 1. The parameter values for Table 1 were chosen to represent a biologically possible system^5^. Here we simulated 1000 virtual experiments and estimated the parameters in each. In each experiment, twelve concentration-response curves corresponding to a pair of reference and test ligands in two signalling pathways (three replicates for every situation) were generated with random error. Then, the dataset generated from every simulation experiment was estimated with the candidate model. For each simulation experiment, we calculated the Wald statistic (the ratio of squared ΔΔ*logR*_1−2_ estimate and its variance, $\frac{{\hat{\theta }}_{{\rm{\Delta }}{\rm{\Delta }}log{R}_{1-2}}^{2}}{s{e}^{2}({\hat{\theta }}_{{\rm{\Delta }}{\rm{\Delta }}log{R}_{1-2}})}$). The 95^th^ percentile from 1000 Wald statistics was used as the cut-off criterion for type I error (α) equal to 0.05.

**Table 1:** Parameter values and study design for the calibration of alpha criterion (*α* = 0.05).

Parameters	Reference	Test
**Design**		
*logA*	From −11 to −4, increase by 1	
**Pathway 1**		
*E*_*m*1_	100	100
*Basal*_1_	10	10
*logR*_1_	7	6
**Pathway 2**		
*E*_*m*2_	100	100
*Basal*_2_	10	10
*logR*_2_	7	6
$log{K}_{A}^{^{\prime} }$	−5	−6
ΔΔ*logR*_1−2_	0	
**Random error**		
*add*.*err*_1_	5	5
*prop*.*err*_1_	10%	10%
*add*.*err*_2_	5	5
*prop*.*err*_2_	10%	10%
2. Calculation of power curve

The pseudo-experimental data were simulated from the true model (*i.e*., the intact operational model) under alternative hypothesis (H_0_: ΔΔ*logR*_1−2_ ≠ 0), based on the given parameter values and study design in Table 2 (the same as Table 1, except that ΔΔ*logR*_1−2_ was different from zero). For each ΔΔ*logR*_1−2_ value, we conducted 1000 simulation and estimation experiments. In each experiment, twelve concentration-response curves corresponding to a pair of reference and test ligands in two signalling pathways (three replicates for every situation) were generated with random error. Then, the dataset generated from every simulation experiment was estimated with both candidate models (the marginal and intact operational models). For each simulation experiment, we calculated Wald statistic $(\frac{{\hat{\theta }}_{{\rm{\Delta }}{\rm{\Delta }}log{R}_{1-2}}^{2}}{s{e}^{2}({\hat{\theta }}_{{\rm{\Delta }}{\rm{\Delta }}log{R}_{1-2}})})$. For each ΔΔ*logR*_1−2_ value, the power was calculated by taking the percentage of Wald statistics that were greater than the criterion for type I error equal to 0.05. Since, in this experiment, ligand bias was present then the proportion of statistically significant runs is an empirical approximation to the true power.

**Table 2:** Parameter values and study design for the calculation of power curve.

Parameters	Reference	Test
**Design**		
*logA*	From −11 to −4, increase by 1	
**Pathway 1**		
*E*_*m*1_	100	100
*Basal*_1_	10	10
*logR*_1_	7	6.1, 6.2, 6.3, 6.4, 6.5, 6.6, 6.8, 7.0
**Pathway 2**		
*E*_*m*2_	100	100
*Basal*_2_	10	10
*logR*_2_	7	6
$log{K}_{A}^{^{\prime} }$	−5	−6
ΔΔ*logR*_1−2_	0.1, 0.2, 0.3, 0.4, 0.5, 0.6, 0.8, 1.0	
**Random error**		
*add*.*err*_1_	5	5
*prop*.*err*_1_	10%	10%
*add*.*err*_2_	5	5
*prop*.*err*_2_	10%	10%

For power analysis of the intact operational model, the intact operational model (Eq. 11) was the candidate model and ${\hat{\theta }}_{{\rm{\Delta }}{\rm{\Delta }}log{R}_{1-2}}$ and $se({\hat{\theta }}_{{\rm{\Delta }}{\rm{\Delta }}log{R}_{1-2}})$ was directly obtained from the output of NONMEM. For power analysis of the marginal operational model, the marginal operational model (Eq. 1) was the candidate model and ${\hat{\theta }}_{{\rm{\Delta }}{\rm{\Delta }}log{R}_{1-2}}$ and $se({\hat{\theta }}_{{\rm{\Delta }}{\rm{\Delta }}log{R}_{1-2}})$ was calculated through *post hoc* analysis (Eqs 2 and 3).

#### Results

**Figure 2:**
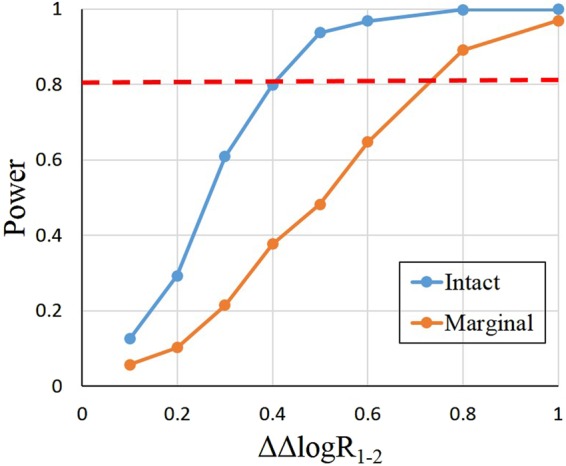
The power curve for the marginal operational model and the intact operational model. The orange line indicates the power curve for the marginal operational model and the blue line indicates the power curve for the intact operational model. The red dashed line indicates 80% power.

The cut-off criterion for type I error equal to 0.05 was 5.53 for the intact operational model and 3.67 for the marginal operational model. The theoretical criterion was 3.84. For intact operational model, there was a slight increase in the type I error rate. These cut-off criterion values were used in the power analysis for controlling of type I error. The power analysis results are presented in Fig. 2. It is seen that the intact operational model had greater power to detect ligand bias compared to the marginal operational model for all values of ΔΔ*logR*_1−2_. It was shown that ΔΔ*logR*_1−2_ had to be greater than 0.8 for the marginal operational model to achieve 80% power for the ligand bias, while, for the intact operational model, the requirement of ΔΔ*logR*_1−2_ was approximately 0.4.

## Part III

### Application of the intact operational model

The performance of the intact operational model was evaluated using two examples from the literature. In both examples signalling responses were measured, simultaneously, a desirable but not necessary characteristic for application of the intact model (to reduce assay-specific effects). In the first example, calculation of *logR* for the marginal operational model does not support continued experiments since *logR* could not be precisely determined for the single ligand under the marginal operation model, whereas estimation was sufficiently precise under the intact model to support a full analysis. In the second example, we work through to calculation of ΔΔ*LogR* and arrive at a refined (and different) conclusion from the intact operational model.

#### Example I: the opposing effects of G_i_ and G_s_ signalling

Data description: In example I, data were extracted from the literature that described the opposing effects of G_i_ and G_s_ signalling pathways on α_2_-adrenergic receptor subtype C10 mediated adenylate cyclase activity in transfected Chinese hamster ovary (CHO) cells expressing different levels of receptors^14^. At low expression levels (1 pmol/mg), only the inhibitory effect of the G_i_ pathway was observed; at higher receptor levels (10 pmol/mg), an atypical biphasic response was observed due to stimulatory effect of G_s_ pathway.

Model analysis: Marginal operational model: In this example the two signalling pathways (Gi and Gs) resulted in a change in the same pharmacological response (cAMP). The marginal operational model was implemented as the sum of the general operational model (Eq. 1) for each pathway (Eq. 12 for low receptor expression level and Eq. 13 for high receptor expression level). With the assumption that each signalling pathway was independent, the marginal operational model was implemented without any constraint on the functional affinity in different pathways. Hence, *K*_*A*_ could be different in G_i_ and G_s_ pathways, denoted as *K*_*A*,*i*_ and *K*_*A*,*s*_.

12 $${E}^{L}=Basal-\frac{{E}_{m,i}}{1+{(\frac{(\frac{A}{{10}^{log{K}_{A,i}}}+1)}{{10}^{log{R}_{i}^{L}}\cdot A})}^{{n}_{i}}}\,+\frac{{E}_{m,s}}{1+{(\frac{(\frac{A}{{10}^{log{K}_{A,s}}}+1)}{{10}^{log{R}_{s}^{L}}\cdot A})}^{{n}_{s}}}$$

13 $${E}^{H}=Basal-\frac{{E}_{m,i}}{1+{(\frac{(\frac{A}{{10}^{log{K}_{A,i}}}+1)}{{10}^{log{R}_{i}^{H}}\cdot A})}^{{n}_{i}}}\,+\frac{{E}_{m,s}}{1+{(\frac{(\frac{A}{{10}^{log{K}_{A,s}}}+1)}{{10}^{log{R}_{s}^{H}}\cdot A})}^{{n}_{s}}}$$

The subscript ‘i’ and ‘s’ indicates the G_i_ and G_s_ pathways, respectively. The superscript ‘L’ and ‘H’ indicates the low receptor expression level and high receptor expression level, respectively. We note here that the basal level of activity is common to both pathways.

By definition of the transduction coefficient (*R*) and the transducer ratio (*τ*), it was demonstrated that the transduction coefficient was proportional to the total receptor density (*R*_*t*_) (Eq. 14).

14 $$R=\frac{\tau }{{K}_{A}}=\frac{\frac{{R}_{t}}{{K}_{E}}}{{K}_{A}}=\frac{{R}_{t}}{{K}_{E}\cdot {K}_{A}}$$

Hence, the relationship between transduction coefficients in the G_i_ pathway at two receptor expression levels (1 pmol/mg and 10 pmol/mg) could be established (Eqs 15 and 16).

15 $$\frac{{R}_{i}^{H}}{{R}_{i}^{L}}=\frac{{R}_{t}^{H}}{{R}_{t}^{L}}=\frac{10\,pmol/mg}{1\,pmol/mg}=10$$

16 $$log{R}_{i}^{H}=log{R}_{i}^{L}+1$$

Similarly, the relationship between transduction coefficients in the G_s_ pathway at two receptor expression levels (1 pmol/mg and 10 pmol/mg) was also established (Eq. 17).

17 $$log{R}_{s}^{H}=log{R}_{s}^{L}+1$$

Then, substituting Eqs 16 and 17 into Eq. 13 yields the equation for quantifying pharmacological response at high receptor level (Eq. 18).

18 $${E}^{H}=Basal-\frac{{E}_{m,i}}{1+{(\frac{(\frac{A}{{10}^{log{K}_{A,i}}}+1)}{{10}^{(log{R}_{i}^{L}+1)}\cdot A})}^{{n}_{i}}}\,+\frac{{E}_{m,s}}{1+{(\frac{(\frac{A}{{10}^{log{K}_{A,s}}}+1)}{{10}^{(log{R}_{s}^{L}+1)}\cdot A})}^{{n}_{s}}}$$

In this case, Eqs 12 and 18 were used to model the data from Example I, where; *logK*_*A*,*i*_, *logK*_*A*,*s*_, $log{R}_{i}^{L}$ and $log{R}_{s}^{L}$ were the drug specific parameters that would be directly estimated.

A preference of a ligand towards a particular pathway before normalisation to a reference ligand was termed the ligand preference profile. It could be regarded as an intermediate metric from the first normalisation process for calculating ligand bias metric (detailed in Appendix 3). Hence, the ligand preference profile between G_i_ and G_s_ signalling pathways was defined as Eq. 19:

19 $$log{R}_{i:s}=log{R}_{i}^{L}-log{R}_{s}^{L}$$

For the marginal operational model, the ligand preference profile (*logR*_*i*:*s*_) was calculated using Eq. 19 and the estimated standard error (SE) was calculated using Eq. 20:

20 $$S{E}_{(log{R}_{i:s})}=\sqrt{S{E}_{(log{R}_{i}^{L})}^{2}+S{E}_{(log{R}_{s}^{L})}^{2}}$$

Intact operational model: The intact operational model was implemented as Eq. 21 for low receptor level and Eq. 22 for high receptor level. Note here that the apparent equilibrium dissociation constant (${K}_{A}^{^{\prime} }$) was shared by G_i_ and G_s_ pathways.

21 $${E}^{L}=Basal-\frac{{E}_{m,i}}{1+{(\frac{(\frac{A}{{10}^{log{K}_{A}^{^{\prime} }}}+1)}{{10}^{log{R}_{i}^{L}}\cdot A})}^{{n}_{i}}}\,+\frac{{E}_{m,s}}{1+{(\frac{(\frac{A}{{10}^{log{K}_{A}^{^{\prime} }}}+1)}{{10}^{log{R}_{s}^{L}}\cdot A})}^{{n}_{s}}}$$

22 $${E}^{H}=Basal-\frac{{E}_{m,i}}{1+{(\frac{(\frac{A}{{10}^{log{K}_{A}^{^{\prime} }}}+1)}{{10}^{log{R}_{i}^{H}}\cdot A})}^{{n}_{i}}}\,+\frac{{E}_{m,s}}{1+{(\frac{(\frac{A}{{10}^{log{K}_{A}^{^{\prime} }}}+1)}{{10}^{log{R}_{s}^{H}}\cdot A})}^{{n}_{s}}}$$

Based on the definition of ligand preference between G_i_ and G_s_ signalling pathways (Eq. 19), $log{R}_{s}^{L}$ are expressed as the difference of $log{R}_{i}^{L}$ and *logR*_*i*:*s*_ (Eq. 23):

23 $$log{R}_{s}^{L}=log{R}_{i}^{L}-log{R}_{i:s}$$

Substituting Eq. 23 into Eq. 17 yielded the expression of $log{R}_{s}^{H}$ (Eq. 24):

24 $$log{R}_{s}^{H}=log{R}_{i}^{L}-log{R}_{i:s}+1$$

Then, substituting Eqs 16, 23 and 24 into Eqs 21 and 22 yielded the intact operational model with *logR*_*i*:*s*_ as a directly estimated parameter (Eq. 25 for low receptor level and Eq. 26 for high receptor level).

25 $${E}^{L}=Basal-\frac{{E}_{m,i}}{1+{(\frac{(\frac{A}{{10}^{log{K}_{A}^{^{\prime} }}}+1)}{{10}^{log{R}_{i}^{L}}\cdot A})}^{{n}_{i}}}\,+\frac{{E}_{m,s}}{1+{(\frac{(\frac{A}{{10}^{log{K}_{A}^{^{\prime} }}}+1)}{{10}^{(log{R}_{i}^{L}-log{R}_{i:s})}\cdot A})}^{{n}_{s}}}$$

26 $${E}^{H}=Basal-\frac{{E}_{m,i}}{1+{(\frac{(\frac{A}{{10}^{log{K}_{A}^{^{\prime} }}}+1)}{{10}^{(log{R}_{i}^{L}+1)}\cdot A})}^{{n}_{i}}}\,+\frac{{E}_{m,s}}{1+{(\frac{(\frac{A}{{10}^{log{K}_{A}^{^{\prime} }}}+1)}{{10}^{(log{R}_{i}^{L}-log{R}_{i:s}+1)}\cdot A})}^{{n}_{s}}}$$

In this case, $log{K}_{A}^{^{\prime} }$, $log{R}_{i}^{L}$ and *logR*_*i*:*s*_ were the ligand specific parameters. Hence, the profile metric (*logR*_*i*:*s*_) is estimated directly and the estimated standard error can be obtained from the NONMEM modelling output.

#### Results

**Figure 3:**
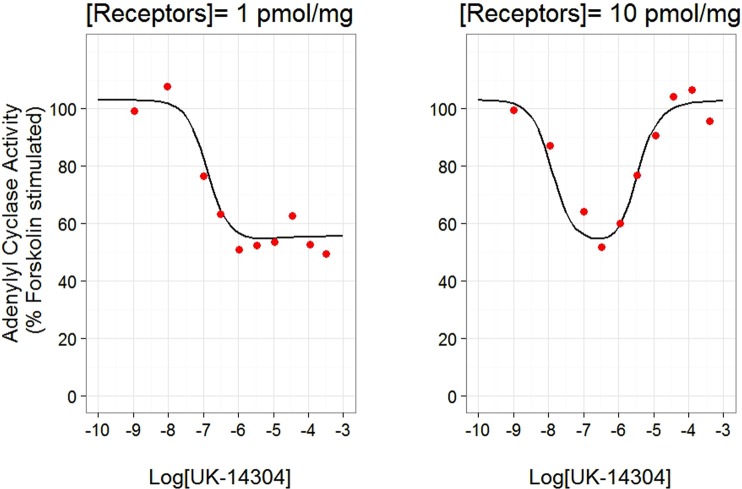
The fitting result of the intact operational model for the effect of the α2-adrenoceptor agonist UK-14304 on adenylate cyclase in transfected CHO cells expressing α2-C10 receptors at 1.0 and 10.0 pmol/mg. Note, G_i_ and G_s_ pathways are acting under both conditions, however the G_i_ pathway dominates at the low receptor expression and G_s_ pathway can eventually overcome this at high receptor expression. Left: at expression level of 1.0 pmol/mg, G_i_-protein mediated inhibition of adenylate cyclase played a dominant role. Right: at expression level of 10 pmol/mg, a ‘U-shape’ response was observed due to activation of G_s_-protein. The red dots are the data grabbed from the literature^14^. The black lines are the simulation profiles from the intact operational model.

As illustrated in Fig. 3, the prediction from the intact operational model could reproduce the effect of the α_2_-adrenoceptor agonist UK-14304 on adenylate cyclase in transfected CHO cells, both qualitatively and quantitatively. This was confirmed by the model evaluation plots (Figures S1 and S2).

From Table 3, the estimated parameter values were generally comparable between the intact operational model and the marginal operational model. However, it was evident that the intact operational model rendered more precise parameter estimates than the marginal operational model. Some parameters (*e.g*., *E*_*m*,*s*_ and *n*_2_) from the marginal operational model yielded poorly estimated parameters with relative standard errors of more than 200%, indicating that the data (from a standard pharmacological experiment) was not sufficiently informative to allow precise estimation of these parameters. In particular, the ligand preference profile (*logR*_*i*:*s*_ = 2.22), calculated via a *post hoc* analysis from the marginal operational model, was imprecise with relative standard error of 69%. Given this level of imprecision there would be little value in continuing with further comparator ligands since discrimination between ligands is unlikely to be fruitful. Hence, by testing only one ligand and analysing it in this way it is possible to make a no-go decision until an improved experimental set up could be created. In contrast, with the same profile directly estimated from the intact operational model was much more precise with the relative standard error of 6.7%. Hence, it remains possible to detect ligand bias in upcoming experiments and further refinement of the experiment would be unnecessary if the intact operational model is used.

**Table 3:** Comparison of the estimation results from the intact operational model and the marginal operational model for the effect of α_2_-adrenoceptor agonist UK-14304 on CHO cells with different expression levels of α_2_C10 receptor.

Parameters	Intact operational model	Marginal operational model
	Estimated [RSE%]	
*Basal*	103 [2.9%]	103 [2.9%]
*E*_*m*, *i*_	49.2 [8.8%]	48.7 [16.2%]
*n*_*i*_	1.47 [48.2%]	1.44 [52.0%]
*E*_*m*, *s*_	59.2 [23.3%]	69.9 [212%]
*n*_*s*_	2.27 [28.7%]	2.54 [302%]
$log{K}_{A}^{^{\prime} }$	−5.32 [3.9%]	—
*logK*_*A*, *i*_	—	0 FIX^a^
*logK*_*A*, *s*_	—	−5.54 [57.6%]
$log{R}_{i}^{L}$	—	6.90 [1.7%]
$log{R}_{s}^{L}$	—	4.68 [32.5%]
*logR*_*i*: *s*_	2.28 [6.7%]	2.22 [68.7%]^b^

RSE, relative standard error.

A subscript letter ‘i’ indicates the G_i_ signalling pathway, ‘s’ indicates the G_s_ signalling pathway and ‘L’ indicates the lower receptor expression.

a: α_2_-adrenoceptor agonist UK-14304 appears to be full agonist of Gi pathways in both receptor expression levels (1 pmol/mg and 10 pmol/mg). Due to a deterministic identifiability issue of the marginal operational model, it is not possible to estimate *logK*_*A*_ values for full agonists from direct fitting to concentration-response curve. Conventionally, *logK*_*A*_ is arbitrarily fixed to 0 to solve this problem^3^.

b: calculated from *post hoc* analysis.

#### Example II: simultaneous measurement of IP accumulation and AA release

Data description: Data from the simultaneous measurement of phospholipase C (PLC)-mediated inositol phosphate (IP) accumulation and phospholipase A_2_ (PLA_2_)-mediated arachidonic acid (AA) release after the activation of 5-HT_2C_ receptors in the CHO-1C19 cell were extracted from^15^.

Model analysis: For the marginal operational model, Eq. 1 was separately applied to each signalling pathway to fit the data. For the intact operational model, Eq. 11 was implemented to jointly model all the functional assay data. In order to circumvent the deterministic identifiability issue in marginal operational model, we followed the convention to arbitrarily set $log{K}_{A1}^{TFMPP}$ and $log{K}_{A2}^{BUF}$^11^.

The high efficacy ligand in both pathways, bufotenin (BUF), was chosen as the reference ligand^7^. For the marginal operational model, the ligand bias metric (ΔΔ*logR*_1−2_) and the estimated standard error were calculated via *post hoc* analysis (Eqs 2 and 3). For the intact operational model, the ligand bias metric was directly estimated and the estimated standard error could be obtained from NONMEM modelling output.

Test for ligand bias: The null hypothesis for this test was that ΔΔ*logR*_1−2_ was equal to 0. Since there were four comparisons in this example, the α value was adjusted to 0.0125.

#### Results

**Figure 4:**
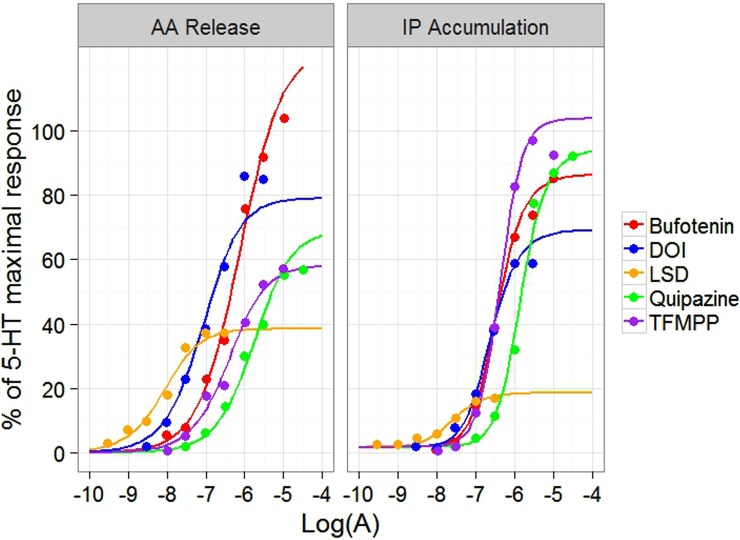
The fitting result of the intact operational model for the effect of the 5-HT_2C_ receptor agonists on PLA2-mediated AA release (left) and PLC-mediated IP accumulation (right) in CHO-1C19 cells. All the responses have been normalized to 5-HT maximal response. The dots are the data grabbed from the literature^15^. The lines are the simulation profiles from the intact operational model. Here, different colours indicate different types of ligands. Red: Bufotenin. Blue: DOI, (±)-1-(2,5-dimethoxy-4-iodophenyl)-2-aminopropane. Yellow: LSD, lysergic acid diethylamide. Green: Quipazine. Purple: TFMPP, 3-trifluoromethylphenyl-piperazine.

As shown in Fig. 4, the intact operational model captured the signalling profiles of the 5-HT_2C_ receptor agonists on PLA_2_-mediated AA release and PLC-mediated IP accumulation in CHO-1C19 cells. This was confirmed by the model evaluation plots (Figs S3 and S4).

From Table 4, the parameter estimates from the intact operational model were comparable to the marginal operational model. Since the *logR* could be precisely estimated in the marginal operational model, considered as a go from a go/no-go decision, we moved forward to the full ligand bias analysis. It was noted that the ligand bias metrics calculated via *post hoc* analyses from the marginal operational model were generally less precise than those directly estimated from the intact operational model. For instance, the relative standard error of ligand bias for QUI from the marginal operational model was almost 400%, much higher than that from the intact operational model (41%). For the test of ligand bias (Table 5), the results of two ligands (QUI, and TFMPP) were comparable from both methods and no statistically significant bias would be concluded for both QUI and TFMPP. However, the intact operational model was able to identify two biased ligands (DOI and LSD) that were not classified as biased ligands by the marginal operational model.

**Table 4:** Comparison of the estimation results from the intact and marginal operational models for the effect of the 5-HT_2C_ receptor agonists on PLA_2_-mediated AA release and PLC-mediated IP accumulation in CHO-1C19 cells with bufotenin as the reference ligand.

Parameter	Intact operational model					Marginal operational model				
	Estimated [RSE%]									
*Basal*_1_	1.79 [21.2%]					1.83 [22.6%]				
*E*_*m*1_	102 [3.7%]					98 [3.3%]				
*n*_1_	1.53 [5.0%]					1.55 [5.8%]				
*Basal*_2_	0 FIX^a^					0 FIX^a^				
*E*_*m*2_	126 [9.6%]					121 [5.6%]				
*n*_2_	0.80 [6.2%]					0.78 [5.5%]				
	BUF	DOI	LSD	QUI	TFMPP	BUF	DOI	LSD	QUI	TFMPP
$log{K}_{A}^{^{\prime} }$	−6.05 [1.2%]	−6.46 [1.4%]	−7.77 [0.9%]	−5.24 [2.7%]	−5.97 [3.2%]	—	—	—	—	—
*logK*_*A*1_	—	—	—	—	—	−6.02 [1.4%]	−6.52 [1.0%]	−7.81 [0.9%]	−4.72 [12.1%]	0 FIX^b^
*logK*_*A*2_	—	—	—	—	—	0 FIX^b^	−5.98 [2.8%]	−7.66 [1.3%]	−5.45 [1.3%]	−5.86 [2.8%]
*logR*_1_	6.50 [0.5%]	6.65 [0.7%]	7.31 [1.0%]	5.89 [1.0%]	6.37 [0.4%]	6.51 [0.5%]	6.70 [0.6%]	7.37 [1.1%]	5.85 [0.8%]	6.39 [0.4%]
*logR*_2_	6.11 [2.2%]	—	—	—	—	6.14 [1.8%]	6.65 [1.2%]	7.28 [1.6%]	5.44 [1.8%]	5.85 [2.2%]
ΔΔ*logR*_1−2_	—	−0.476 [14.3%]	−0.393 [14.4%]	0.167 [40.7%]	0.102 [131%]	—	−0.32 [45.6%]^c^	−0.28 [65.3%]^c^	−0.04 [396%]^c^	−0.17 [103%]^c^

RSE, relative standard error; CV, coefficient of variation; Add. error, additive error; BUF, bufotenin; DOI, (±)-1-(2,5-dimethoxy-4-iodophenyl)-2-aminopropane; LSD, lysergic acid diethylamide; QUI, quipazine; TFMPP, 3-trifluoromethylphenyl-piperazine.

A subscript number “1” indicates the IP accumulation pathway and “2” indicates the AA release pathway.

a: The estimated basal effect of AA release pathway was not significantly different from 0. Therefore, it was fixed to 0 in the model refinement process to make the model more stable.

b: TFMPP appears to be full agonist of IP accumulation pathway and bufotenin appears to be full agonist of AA release pathway. Due to the identifiability issue of the marginal operational model, it is not possible to estimate *logK*_*A*_ values for full agonists from direct fitting to concentration-response curve from that pathway. Conventionally, *logK*_*A*_ is arbitrarily fixed to 0 to solve this problem^3^.

c: calculated from *post hoc* analyses

**Table 5:** Test of ligand bias of 5-HT_2C_ receptor agonists on PLA_2_-mediated AA release and PLC-mediated IP accumulation in CHO-1C19 cells with bufotenin as the reference.

ΔΔ*logR*_1−2_	Intact operational model		Marginal operational model	
	Estimated [RSE%]	p-value	Estimated [RSE%]	p-value
DOI	−0.476 [14.3%]	<0.0125	NS	0.028
LSD	−0.393 [14.4%]	<0.0125	NS	0.126
QUI	NS	0.014	NS	0.800
TFMPP	NS	0.447	NS	0.330

DOI, (±)-1-(2,5-dimethoxy-4-iodophenyl)-2-aminopropane; LSD, lysergic acid diethylamide; QUI, quipazine; TFMPP, 3-trifluoromethylphenyl-piperazine.

A subscript number “1” indicates the IP accumulation pathway and “2” indicates the AA release pathway.

NS indicates no statistically significant bias.

## Discussion

In this work, an intact version of the operational model was proposed as a more complete representation of the biological system. The intact operational model follows the concept of functional selectivity by explicitly implementing linked equilibria among different receptor conformational states. Hence, the apparent dissociation constant (${K}_{A}^{^{\prime} }$) is the same among different signalling pathways to reflect the natural correlation of *C*_50_ among different pathways (Figure S5). The intact operational model allows for joint modelling of all data from different pathways and provides a direct estimate of ΔΔ*logR*, which avoids propagation of errors in the *post hoc* analysis. At a practical level this means the bias parameters (*i.e*. ΔΔ*logR*) are more precisely estimated for any given experimental design and the intact operational model has greater power (more sensitivity) to detect weak ligand bias that might otherwise be missed by the marginal operational model. This latter feature provides the potential for more in-depth inferences from experiments.

According to the power analysis evaluation, both marginal and intact operational model work well for highly biased ligands (ΔΔ*logR* > 1), with the power for ligand bias test exceeding 80%. However, only the intact operational model is able to detect less strongly biased ligands (ΔΔ*logR* < 0.8) with confidence (power exceeding 80%). The result from this theoretical evaluation is consistent with the finding from one previous study^16^. It is apparent that the marginal operational model fails to distinguish true ligand bias from ‘error’ with confidence, especially when the ligand bias is weak. Our work further supports this finding and provides statistical and mechanistic support for the intact model in detecting ligand bias.

The concept of approaching a pharmacology experiment within a go/no-go workflow framework was demonstrated in current work. In Example I, it would be plausible to consider a no-go decision based on the fact that *logR*_*i*:*s*_ could not be precisely determined for the single ligand when using the marginal operational model and hence the future ability to demonstrate ligand bias would therefore be unlikely. In this case, it would be necessary for the investigator to reconsider the experimental conditions. However, application of the intact operational model to the same data yielded a precise estimate of the ligand preference profile *logR*_*i*:*s*_ and hence would be anticipated to support further work to estimate full ligand bias (ΔΔ*logR*) from the current experimental set up. In Example II, the values of *logR* could be precisely estimated with the marginal operational model and hence a logical go decision would be made. We see however, in this example that even working through the full experiment the marginal operational model did not identify bias in some pathways when the intact operational model did.

Though the intact operational model is a more mechanism-based model framework for functional selectivity, it is not as flexible as the marginal operational model in curve fitting. In the operational model, for low efficacy agonists (*i.e*., as *τ* approaches 0), the *C*_50_ approximates *K*_*A*_ (Eq. 27). As the values of *K*_*A*_ are the same for different signalling pathways (all equal to ${K}_{A}^{^{\prime} }$) in the intact operational model, this precludes fitting concentration–response curves of a ligand behaving as partial agonists in two pathways with very different *C*_50_ values which would require equally distinct *K*_*A*_^17^. In addition, an exact numerical evaluation (detailed in Appendix 4) was performed in a case with a non-unity slope factor, showing that the difference between *C*_50_ values could not exceed (approximately) 100 fold. Such a change in *C*_50_ values is unlikely to occur biologically, but rather, when this is observed it likely reflects that the responses of different signalling pathways are commonly measured under different experimental conditions, which may break the natural linkage among different signalling pathways.

27 $${C}_{50}=\frac{{K}_{A}}{\tau +1}\mathop{\to }\limits^{\tau \to 0}\frac{{K}_{A}}{1}$$

Another model for quantifying functional selectivity is Rajagopal’s model^18–20^. In Rajagopal’s model, the operational model is separately applied to each signalling pathway with the constraint that *K*_*A*_ among different pathways are all fixed to a previously estimated value from a separate binding assay. This model shares the same mathematical form with the intact operational model (detailed in Appendix 5). Hence, the intact operational model could be regarded as one possible biological interpretation for Rajagopal’s model. However, it is noted that in Rajagopal’s model, this shared functional affinity is fixed rather than determined by the data^18^. The intact operational model does not require this constraint. Whether functional affinity should be fixed to a measured value from another binding assay or estimated as part of the current experimental set up is out of the scope of current work. But it is worth noting that most binding assays are performed on cell membranes, while signalling assays are performed on whole cells and therefore may not be representative of each other. One possible strategy would be to use a fully Bayesian approach with informative priors on parameters that would have otherwise been fixed. More theoretical and practical evaluations are warranted to address this.

The three-state model is another mechanism-based model for quantifying function selectivity^8,15^. Similar to the intact operational model, the linked equilibria among different receptor conformation states and the mutual depletion of these receptor states are explicitly implemented in the three-state model. Within this model framework, the *EC*_50_ values are the same for different signalling pathways, which makes it less flexible than the intact operational model. Moreover, the three-state model only accounts for the receptor binding and does not include signal transduction processes. Hence, it cannot explain the phenomenon often termed ‘receptor reserve’, and no ligand may behave as a full agonist in both pathways. Additionally, given only functional assay data, the three-state model is not structurally identifiable and only a simulation-based, heuristic search for parameter values is possible (see for example^15^), which limits its utility in quantifying functional selectivity. Contrary to the empirical basal effect in the intact operational model, the three-state model mechanistically incorporates constitutive activity. In the future, it is desirable to combine these two model structures to render more insight into the underlying mechanism of functional selectivity.

As demonstrated in this work, the intact operational model works well when different signalling pathways are measured simultaneously. However, in practice, it is not uncommon that the responses from different signalling pathways are measured under quite different experimental conditions. The experimental conditions for the functional assay to study one pathway may eliminate the responses from other pathways. Under this circumstance, the association between different active receptor states can be disentangled and the intact operational model can be reduced into the marginal operational model (Appendix 6). In this sense, the marginal operational model can be regarded as a special case of the intact operational model when the responses from different pathways are measured under different conditions. However, in order to gain the maximum mechanistic insight into functional selectivity by application of the intact operational model, it is desirable that the links between different signalling pathways are preserved by keeping experimental conditions as consistent as possible between assay types.

For the accessibility of the intact operational model, our primary goal was to implement it in GraphPad Prism (a standard software in the area of experimental pharmacology). However, due to the nature of the model this was not possible within Prism. In this work, we used a more general and flexible analysis platform, in this case NONMEM. Though NONMEM has been widely used in the area of pharmacometrics^21^, there are only few applications in analytical pharmacology^22^. There are other platforms like NONMEM offering flexibility in modelling, such as Monolix (developed by Lixoft) and Phoenix NLME (developed by Cetara), which could be used for analysis of this sort of data. (We provide the NONMEM estimation control streams as a Supplement).

In conclusion, the intact operational model can be applied to circumstances where the marginal operational model has been used. The intact operational model is more sensitive to identifying biased ligands (i.e. has greater power), and provides a more precise estimate of the operational model ligand bias metric (ΔΔ*logR*). The intact operational model may provide a valuable step to describe and improve understanding of the underlying mechanisms of functional selectivity.

## Supplementary information

Appendix

Supplemental Information

## Data Availability

All data analysed during this study are collected from published literatures.

## References

[1] Urban JD (2007). Functional selectivity and classical concepts of quantitative pharmacology. J Pharmacol Exp Ther.

[2] Winpenny D, Clark M, Cawkill D (2016). Biased ligand quantification in drug discovery: from theory to high throughput screening to identify new biased mu opioid receptor agonists. Br J Pharmacol.

[3] van der Westhuizen ET, Breton B, Christopoulos A, Bouvier M (2014). Quantification of ligand bias for clinically relevant β2-adrenergic receptor ligands: implications for drug taxonomy. Mol Pharmacol.

[4] Black JW, Leff P (1983). Operational models of pharmacological agonism. Proc R Soc Lond B Biol Sci.

[5] Kenakin T, Watson C, Muniz-Medina V, Christopoulos A, Novick S (2012). A simple method for quantifying functional selectivity and agonist bias. ACS Chem Neurosci.

[6] Kenakin T, Christopoulos A (2013). Signalling bias in new drug discovery: detection, quantification and therapeutic impact. Nat Rev Drug Discov.

[7] Klein Herenbrink C (2016). The role of kinetic context in apparent biased agonism at GPCRs. Nat Commun.

[8] Leff P, Scaramellini C, Law C, McKechnie K (1997). A three-state receptor model of agonist action. Trends Pharmacol Sci.

[9] Beal, S. L., Sheiner, L. B., Boeckmann, A. & Bauer, R. J. NONMEM users guides. *NONMEM Project Group, University of California, San Francisco* (1992).

[10] Keizer RJ, Karlsson MO, Hooker A (2013). Modeling and Simulation Workbench for NONMEM: Tutorial on Pirana, PsN, and Xpose. CPT Pharmacometrics Syst Pharmacol.

[11] Zhu, X., Finlay, D. B., Glass, M. & Duffull, S. B. An evaluation of the operational model when applied to quantify functional selectivity. *Br J Pharmacol*, 10.1111/bph.14171 (2018). 10.1111/bph.14171 PMC5913411 29457969

[12] Wang YN (2007). Derivation of various NONMEM estimation methods. J Pharmacokinet Pharmacodyn.

[13] Dunn, O. J. Multiple Comparisons among Means. *J Am Stat Assoc* **56**, 52-&, 10.2307/2282330 (1961).

[14] Eason MG, Kurose H, Holt BD, Raymond JR, Liggett SB (1992). Simultaneous coupling of alpha 2-adrenergic receptors to two G-proteins with opposing effects. Subtype-selective coupling of alpha 2C10, alpha 2C4, and alpha 2C2 adrenergic receptors to Gi and Gs. J Biol Chem.

[15] Berg KA (1998). Effector pathway-dependent relative efficacy at serotonin type 2A and 2C receptors: Evidence for agonist-directed trafficking of receptor stimulus. Mol Pharmacol.

[16] Onaran HO (2017). Systematic errors in detecting biased agonism: Analysis of current methods and development of a new model-free approach. Sci Rep.

[17] Kenakin T, Christopoulos A (2013). Measurements of ligand bias and functional affinity. Nat Rev Drug Discov.

[18] Rajagopal S (2011). Quantifying Ligand Bias at Seven-Transmembrane Receptors. Mol Pharmacol.

[19] Rajagopal S (2013). Quantifying biased agonism: understanding the links between affinity and efficacy. Nat Rev Drug Discov.

[20] Onaran HO, Rajagopal S, Costa T (2014). What is biased efficacy? Defining the relationship between intrinsic efficacy and free energy coupling. Trends Pharmacol Sci.

[21] Bauer RJ, Guzy S, Ng C (2007). A survey of population analysis methods and software for complex pharmacokinetic and pharmacodynamic models with examples. AAPS J.

[22] Benson N (2010). Estimation of binding rate constants using a simultaneous mixed‐effects method: application to monoamine transporter reuptake inhibitor reboxetine. Br J Pharmacol.

