# Supplementary material for: An intact model for quantifying functional selectivity
Source: Sci Rep. 2019 Feb 22;9:2557. doi: 10.1038/s41598-019-39000-z (PMC6384912; doi:10.1038/s41598-019-39000-z)
Supplement: Supplementary file 1 — Appendix [file 41598_2019_39000_MOESM1_ESM.pdf]

Appendix for

Title: An intact model for quantifying functional selectivity

Authors: Xiao Zhu[a*], David B. Finlay[b], Michelle Glass[b], Stephen B. Duffull[a]

[a] Otago Pharmacometrics Group, School of Pharmacy, University of Otago, Dunedin, New Zealand

[b] Department of Pharmacology and Clinical Pharmacology, Faculty of Medical and Health Sciences, University of Auckland, Auckland, New Zealand

[*] **Corresponding Author:** Xiao Zhu, derekzx@126.com, phone +6434795045, Otago Pharmacometrics Group, School of Pharmacy, University of Otago, PO Box 56, Dunedin, New Zealand

1. Derivation of the intact operational model

The conventional Black-Leff operational model is extended to explicitly consider functional selectivity. As illustrated in Figure 1, for the intact operational model, the equilibria are linked among different receptor conformation states and there is mutual depletion of these receptor states. Though there may exist a theoretically unlimited number of active states, only two different active receptor conformations are considered in current derivation. The following derivation can be easily generalised into the case of multiple active states.

In the binding process, the formation of active states is jointly governed by the equilibrium dissociation constants K_{A1} and K_{A2}, which determine ligand's affinity for AR^{*} and AR^{**}, respectively:

$$\frac{A \cdot R_{ub}}{AR^{*}} = K_{A1} \qquad (A.1)$$

$$\frac{A \cdot R_{ub}}{AR^{**}} = K_{A2} \qquad (A.2)$$

In the binding process, the receptor is distributed amongst the one unbound (inactive) and two bound (active) states, so the total receptor concentration (R_t) is:

$$R_t = R_{ub} + AR^{*} + AR^{**} \qquad (A.3)$$

Solving the equations leads to the amount of each receptor state:

$$R_{ub} = \frac{R_t}{\left(\frac{1}{K_{A1}} + \frac{1}{K_{A2}}\right) \cdot A + 1} \qquad (A.4)$$

$$AR^{*} = \frac{R_t \cdot A/K_{A1}}{\left(\frac{1}{K_{A1}} + \frac{1}{K_{A2}}\right) \cdot A + 1} \qquad (A.5)$$

$$AR^{**} = \frac{R_t \cdot A/K_{A2}}{\left(\frac{1}{K_{A1}} + \frac{1}{K_{A2}}\right) \cdot A + 1} \qquad (A.6)$$

The stimulus for pathway 1 is given by:

$$S_1 = AR^{*} \qquad (A.7)$$

For the cellular signalling of pathway 1, a rectangular hyperbolic function (Eq. A8) is postulated for the relationship between the stimulus and the observed response.

$$\frac{E_1}{E_{m1}} = \frac{S_1}{S_1 + K_{E1}} \qquad (A.8)$$

Where E_{m1} is the maximal possible response of the pathway 1 and K_{E1} is the concentration of the active receptor state I (AR^*) that produces 50% of the maximal response for pathway 1.

Substituting stimulus with Eq.A5 yields the equation of the intact operational model for pathway 1 (Eq.A9):

$$\frac{E_1}{E_{m1}} = \frac{\frac{\tau_1}{K_{A1}} \cdot A}{\frac{\tau_1}{K_{A1}} \cdot A + \left(\frac{1}{K_{A1}} + \frac{1}{K_{A2}}\right) \cdot A + 1} \qquad (A.9)$$

Here, τ_1 is the term that quantifies the efficacy of the agonist to produce response and the efficiency of stimulus-response coupling. τ_1 is defined as the ratio R_t/K_{E1}. Thus, τ_1 is system-dependent, ligand-dependent and measurement-dependent.

Similarly, the intact operational model for pathway 2 (Eq. A10) is derived:

$$\frac{E_2}{E_{m2}} = \frac{\frac{\tau_2}{K_{A2}} \cdot A}{\frac{\tau_2}{K_{A2}} \cdot A + \left(\frac{1}{K_{A1}} + \frac{1}{K_{A2}}\right) \cdot A + 1} \qquad (A.10)$$

Given pharmacological response data only, the original form of the intact operational model (Eq.A9 and A10) is not structurally identifiable. There are infinite possible combinations of parameters that render the same result. For example, the two parameter sets, ($\tau_1 = 1$, $K_{A1} = 10^{-9}$, $\tau_2 = 5$, $K_{A2} = 10^{-8}$) and ($\tau_1 = 10$, $K_{A1} = 10^{-8}$, $\tau_2 = 0.5$, $K_{A2} = 10^{-9}$), produce exactly identical curves.

To solve the structural identifiability issue related to the original form of the intact operational model, a re-parameterisation is applied. Here, K_A' is denoted as the apparent equilibrium dissociation constant. R_1 and R_2 are denoted as the transduction coefficient (the ratio of transducer ratio and the equilibrium dissociation constant):

$$K_A' = \frac{1}{\frac{1}{K_{A1}} + \frac{1}{K_{A2}}} \qquad (A.11)$$

$$R_1 = \frac{\tau_1}{K_{A1}} \qquad (A.12)$$

$$R_2 = \frac{\tau_2}{K_{A2}} \qquad (A.13)$$

Substituting K'_A and R_1 into Eq.A9 yields the simplified intact operational model for pathway 1:

$$\frac{E_1}{E_{m1}} = \frac{R_1 \cdot A}{R_1 \cdot A + \left(\frac{A}{K'_A} + 1\right)} \qquad (A.14)$$

The equation is then simplified by dividing above and below by $R_1 \cdot A$:

$$\frac{E_1}{E_{m1}} = \frac{1}{1 + \frac{\left(\frac{A}{K'_A} + 1\right)}{R_1 \cdot A}} \qquad (A.15)$$

Similarly, the simplified intact operational model for pathway 2 is derived:

$$\frac{E_2}{E_{m2}} = \frac{1}{1 + \frac{\left(\frac{A}{K'_A} + 1\right)}{R_2 \cdot A}} \qquad (A.16)$$

2. Derivation of the generalised intact operational model

The binding process is the same as before (Eq.A1-A6). For cellular signalling, instead of a rectangular hyperbolic function, a logistic function (Eq. A17) is postulated to describe the relationship between the active receptor state and the observed response.

$$E_1 = \frac{E_{m1} \cdot {AR^*}^{n_1}}{{K_{E1}}^{n_1} + {AR^*}^{n_1}} \qquad (A.17)$$

Where E_{m1} is the maximal possible response of pathway 1 and K_{E1} is the concentration of the active receptor state I (AR^*) that produces 50% of the maximal response for pathway 1.

Substituting Eq. A5 into Eq. A17 yields Eq. A18:

$$E_1 = \frac{E_{m1} \cdot \left(\frac{\tau_1}{K_{A1}}\right)^{n_1} \cdot A^{n_1}}{\left(\frac{\tau_1}{K_{A1}}\right)^{n_1} \cdot A^{n_1} + \left(A \cdot \left(\frac{1}{K_{A1}} + \frac{1}{K_{A2}}\right) + 1\right)^{n_1}} \qquad (A.18)$$

In order to simplify Eq. A18, re-parameterisation is applied. Substituting K_A' and R_1 into Eq.A18 and reorganising yields Eq.A19:

$$E_1 = \frac{E_{m1} \cdot R_1^{n_1} \cdot A^{n_1}}{R_1^{n_1} \cdot A^{n_1} + \left(\frac{A}{K_A'} + 1\right)^{n_1}} \qquad (A.19)$$

The basal response from pathway 1 can be empirically incorporated into Eq. A19 as an ad hoc parameter, $Basal_1$. This yields the generalised intact operational model for pathway 1:

$$E_1 = Basal_1 + \frac{(E_{m1} - Basal_1) \cdot R_1^{n_1} \cdot A^{n_1}}{R_1^{n_1} \cdot A^{n_1} + \left(\frac{A}{K_A'} + 1\right)^{n_1}} \qquad (A.20)$$

The equation is then simplified by dividing above and below by $R_1^{n_1} \cdot A^{n_1}$:

$$E_1 = Basal_1 + \frac{(E_{m1} - Basal_1)}{1 + \left(\frac{\left(\frac{A}{K_A'} + 1\right)}{R_1 \cdot A}\right)^{n_1}} \qquad (A.21)$$

For curve-fitting purposes, the parameters K_A' and R_1 are recast as logarithms (*i.e.*, $10^{logK_A'}$, 10^{logR_1}):

$$E_1 = Basal_1 + \frac{(E_{m1} - Basal_1)}{1 + \left(\frac{\left(\frac{A}{10^{logK_A'}} + 1\right)}{10^{logR_1} \cdot A}\right)^{n_1}} \qquad (A.22)$$

Similarly, the generalised intact model for pathway 2 (Eq. A23) is derived:

$$E_2 = Basal_2 + \frac{(E_{m2} - Basal_2)}{1 + \left(\frac{\left(\frac{A}{10^{logK_A'}} + 1\right)}{10^{logR_2} \cdot A}\right)^{n_2}} \qquad (A.23)$$

3. *The ligand preference profile could be regarded as the first normalisation step in calculating a ligand bias metric*

The *post hoc* computation of ligand bias (the relative preference of a ligand for a particular pathway) consists of two successive normalisation processes. As shown in Eq. A24, a test ligand's transduction coefficient from one pathway is first normalised to that of a reference

ligand from the same pathway in order to accommodate observational bias. This normalised transduction coefficient $\Delta logR$ is then further normalised to that of a second pathway to account for system bias.

$$\Delta\Delta logR_{1-2} = \left(logR_1^{test} - logR_1^{ref}\right) - \left(logR_2^{test} - logR_2^{ref}\right) \qquad (A.24)$$

As demonstrated in Eq.A25, the order of these two normalisation steps is interchangeable. It is mathematically equivalent to first calculate a ligand's preference profile towards a particular pathway and then normalise this profile to that of a reference ligand.

$$\Delta\Delta logR_{1-2} = (logR_1^{test} - logR_2^{test}) - \left(logR_1^{ref} - logR_2^{ref}\right) \qquad (A.25)$$

Here, this preference profile is defined as the difference of a ligand's transduction coefficients in the two pathways (Eq.A26).

$$logR_{1:2} = logR_1 - logR_2 \qquad (A.26)$$

4. The intact operational model cannot account for very different EC_{50} values of a ligand behaving as partial agonists in different pathways

From the generalised operational model, the following relationship is derived (the same as Eq.11 and 12 in the original operational model paper [4]):

$$\frac{E_{max}}{E_m} = \frac{\tau^n}{\tau^n + 1} \qquad (A.27)$$

$$EC_{50} = \frac{K_A}{\sqrt[n]{\tau^n + 2} - 1} \qquad (A.28)$$

Normally, for a partial agonist, its maximal response (E_{max}) will not exceed 80% of maximal system response (E_m). Hence, according to Eq. A27, the range of τ^n is from 0 to 4.

In the intact operational model, the values of K_A are the same for different signalling pathways (all equal to K_A'). Thus, the ratio of EC_{50} in two pathways is derived as Eq. A29:

$$\frac{(EC_{50})_1}{(EC_{50})_2} = \frac{\sqrt[n_2]{\tau_2^{n_2} + 2} - 1}{\sqrt[n_1]{\tau_1^{n_1} + 2} - 1} \qquad (A.29)$$

As the range of τ^n is from 0 to 4, we maximise the ratio by maximising numerator and minimising denominator (Eq. A30):

$$\frac{(EC_{50})_1}{(EC_{50})_2} = \frac{\sqrt[n_2]{\tau_2^{n_2}+2}-1}{\sqrt[n_1]{\tau_1^{n_1}+2}-1} < \frac{\sqrt[n_2]{4+2}-1}{\sqrt[n_1]{0+2}-1} = \frac{\sqrt[n_2]{6}-1}{\sqrt[n_1]{2}-1} \qquad (A.30)$$

For the normal range of n (from 0.5 to 2), the ratio of EC_{50} from a partial agonist in both pathways cannot exceed 84.5 (Eq. A31 and A32).

$$\frac{(EC_{50})_1}{(EC_{50})_2} < \frac{\sqrt[n_2]{6}-1}{\sqrt[n_1]{2}-1} \leq \frac{\sqrt[0.5]{6}-1}{\sqrt[2]{2}-1} = 84.5 \qquad (A.31)$$

$$\frac{(EC_{50})_1}{(EC_{50})_2} < 84.5 \qquad (A.32)$$

5. *The relationship between the intact operational model and Rajagopal's model*

The Eq.A9 and A10 are reorganised by dividing above and below with $\frac{1}{K_{A1}}+\frac{1}{K_{A2}}$:

$$\frac{E_1}{E_{m1}} = \frac{\left(\frac{\frac{\tau_1}{K_{A1}}}{\frac{1}{K_{A1}}+\frac{1}{K_{A2}}}\right)\cdot A}{\left(\frac{\frac{\tau_1}{K_{A1}}}{\frac{1}{K_{A1}}+\frac{1}{K_{A2}}}+1\right)\cdot A+\frac{1}{\frac{1}{K_{A1}}+\frac{1}{K_{A2}}}} \qquad (A.33)$$

$$\frac{E_2}{E_{m2}} = \frac{\left(\frac{\frac{\tau_2}{K_{A2}}}{\frac{1}{K_{A1}}+\frac{1}{K_{A2}}}\right)\cdot A}{\left(\frac{\frac{\tau_2}{K_{A2}}}{\frac{1}{K_{A1}}+\frac{1}{K_{A2}}}+1\right)\cdot A+\frac{1}{\frac{1}{K_{A1}}+\frac{1}{K_{A2}}}} \qquad (A.34)$$

The apparent transducer ratios for pathway 1 (τ_1') and pathway 2 (τ_2') are defined as Eq.A35 and A36, respectively.

$$\tau_1' = \frac{\frac{\tau_1}{K_{A1}}}{\frac{1}{K_{A1}}+\frac{1}{K_{A2}}} \qquad (A.35)$$

$$\tau_2' = \frac{\frac{\tau_2}{K_{A2}}}{\frac{1}{K_{A1}} + \frac{1}{K_{A2}}} \qquad (A.36)$$

Substituting K_A', τ_1' and τ_2' into Eq.A33 and A34 yields the simplified intact operational model for pathway 1 (Eq. A37) and pathway 2 (Eq.A38):

$$\frac{E_1}{E_{m1}} = \frac{\tau_1' \cdot A}{(\tau_1' + 1) \cdot A + K_A'} \qquad (A.37)$$

$$\frac{E_2}{E_{m2}} = \frac{\tau_2' \cdot A}{(\tau_2' + 1) \cdot A + K_A'} \qquad (A.38)$$

It is noted that the intact operational model (Eq.A37 and A38) shares the same mathematical form as Rajagopal's model (Eq.7 in Rajagopal et al., 2011), though the interpretations of model parameters are different. In the intact operational model, functional affinity is constrained to be the same among all the signalling pathways, reflecting the interactions among different active receptor states. In Rajagopal's model, the functional selectivity is not only the same for all the pathways, but also set to the equilibrium dissociation constant of the ligand for the receptor from a separate binding experiment.

6. *Assessment of the relationship between the marginal operational model and the intact operational model when the responses are measured under different conditions*

Under the circumstance that the responses are measured under very different conditions, the relationship between the marginal operational model and the intact operational model is assessed through the case that pathway 2 is perfectly eliminated by the experimental settings for the functional assay of pathway 1(*i.e.*, complete pathway elimination assumption).

When pathway 2 is perfectly eliminated, agonist is much more prone to stabilising AR^* than AR^{**}. In other words, the affinity for AR^* $(1/K_{A1})$ is much higher than that for AR^{**} $(1/K_{A2})$. As conceptually demonstrated in Eq. A39 and A40, with K_{A2} greatly exceeding K_{A1}, the apparent equilibrium dissociation constant K_A' (Eq.4) approaches the equilibrium dissociation constant for pathway 1 (K_{A1}).

$$K_A' = \frac{1}{\frac{1}{K_{A1}} + \frac{1}{K_{A2}}} \xrightarrow{K_{A2} \gg K_{A1}} \frac{1}{\frac{1}{K_{A1}}} \qquad (A.39)$$

$$K'_A = K_{A1} \tag{A.40}$$

Substituting Eq. A40 into Eq.5 yields the intact operational model for pathway 1 with completely eliminated pathway 2 (Eq. A38). This is identical to the marginal operational model for pathway 1 (Eq.1):

$$E_1 = Basal_1 + \frac{(E_{m1} - Basal_1)}{1 + \left(\frac{\left(\frac{A}{10^{logK_{A1}}} + 1\right)}{10^{logR_1} \cdot A}\right)^{n_1}} \tag{A.41}$$

In this sense, the marginal operational model is a special case of the intact operational model when the responses from different pathways are measured under very different conditions.
