# Supplementary material for: An intact model for quantifying functional selectivity
Source: Sci Rep. 2019 Feb 22;9:2557. doi: 10.1038/s41598-019-39000-z (PMC6384912; doi:10.1038/s41598-019-39000-z)
Supplement: Supplementary file 2 — Supplemental Information [file 41598_2019_39000_MOESM2_ESM.pdf]

Supplemental Information for

Title: An intact model for quantifying functional selectivity

Authors: Xiao Zhu[a*], David B. Finlay[b], Michelle Glass[b], Stephen B. Duffull[a]

[a] Otago Pharmacometrics Group, School of Pharmacy, University of Otago, Dunedin, New Zealand

[b] Department of Pharmacology and Clinical Pharmacology, Faculty of Medical and Health Sciences, University of Auckland, Auckland, New Zealand

[*] **Corresponding Author:** Xiao Zhu, derekzx@126.com, phone +6434795045, Otago Pharmacometrics Group, School of Pharmacy, University of Otago, PO Box 56, Dunedin, New Zealand

1. NONMEM code of intact operational model for Example I

```
$PROBLEM Receptor PD

$INPUT C ID LOGA DV RT

;RT: receptor expression level 1 for low, 10 for high

$DATA dat.csv IGNORE=C

$PRED

;Define parameters

BASAL = THETA(1)  ;basal effect

logKA   = THETA(2)  ;equilibrium dissociation constant

EMI   = THETA(3)  ;maximal inhibition effect

logRI = THETA(4)  ;transduction coefficient inhibitory(Gi)

EMS   = THETA(5)  ;maximal stimulation effect

DlogR = THETA(6)  ;logR(Gs)-logR(Gi)

RI = RT*10**logRI

RS = RI*10**(-DlogR)

n1 = THETA(7)     ;Gi

n2 = THETA(8)     ;Gs

;Model part

A = 10**LOGA ;conc of agonist

KA= 10**(logKA)

TAUI=RI*KA

TAUS=RS*KA

IPRED=BASAL-EMI*(TAUI*A)**n1/((KA+A)**n1+(A*TAUI)**n1)+EMS*(TAUS*A)**n2/((KA+A)**n2+(A*TAUS)**n2)

Y=IPRED*(1+ETA(1))+ETA(2)

$THETA

…

$OMEGA

…

$EST MAXEVAL=2000 NOABORT SIG=3 PRINT=5

$COV

; Xpose

$TABLE ID CONC DV RT IPRED CWRES ONEHEADER NOPRINT FILE=sdtab26
```

2. Model Evaluation of Example I

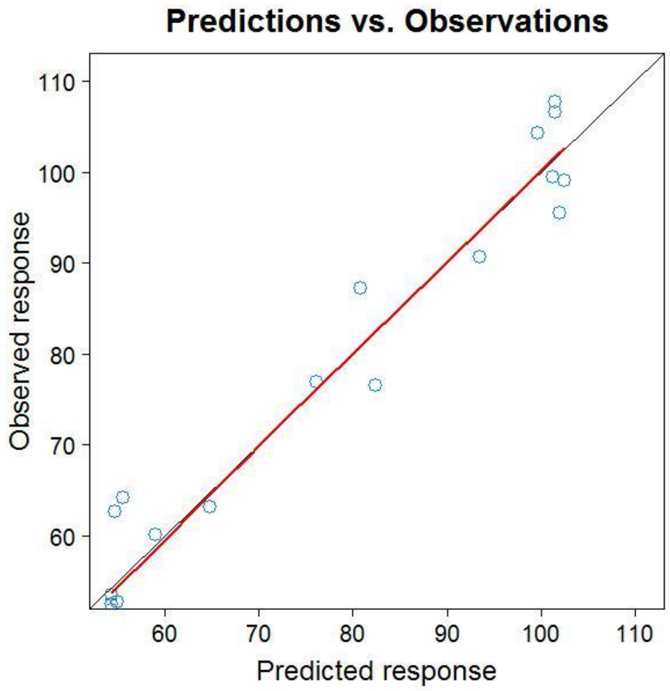

Figure S1. Observed responses vs. predicted responses plot from the intact operational model for Example I.

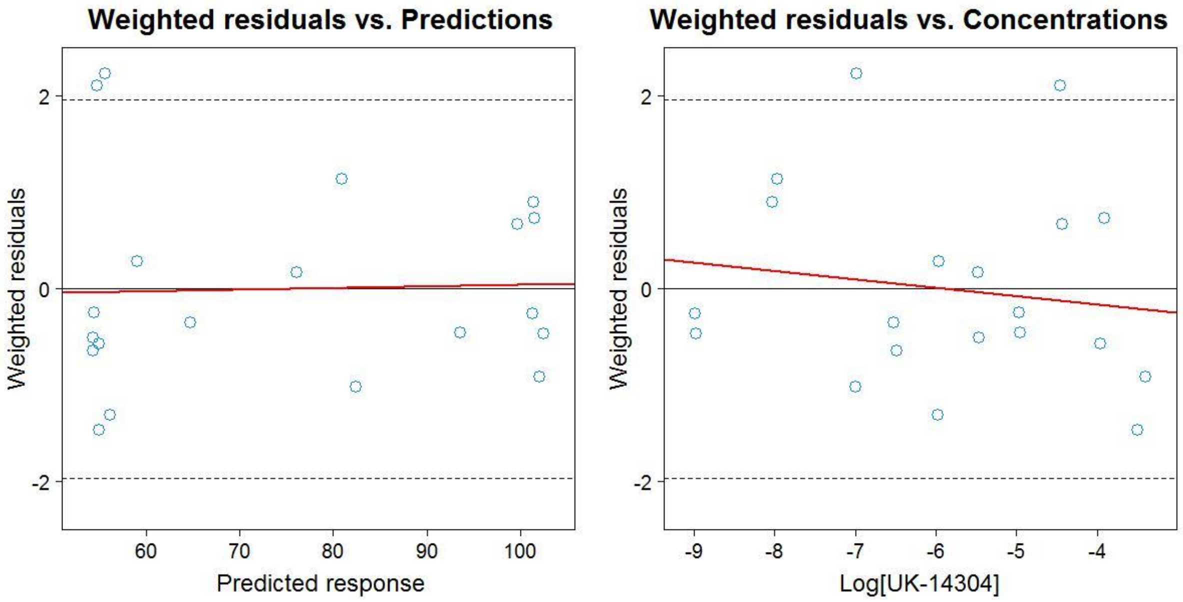

Figure S2. Weighted residuals plots from the intact operational model for Example I. Left: weighted residuals vs. predicted responses. Right: weighted residuals vs. ligand concentrations.

3. NONMEM code of intact operational model for Example II

```
$PROBLEM Receptor PD
$INPUT C ID LOGA DV DRUG TEST
$DATA dat.csv IGNORE=C
$PRED
;Define parameters
BASAL1 = THETA(1)  ;basal effect IP
EM1    = THETA(2)  ;maximal effect IP
BASAL2 = THETA(3)  ;basal effect AA
EM2    = THETA(4)  ;maximal effect AA
;index for different ligands
; 1:bufotenin; 2:DOI; 3.LSD; 4.quipazine; 5.TFMPP
IDX1   = 0
if (DRUG.EQ.1) IDX1 = 1 ;BUF
IDX2   = 0
if (DRUG.EQ.2) IDX2 = 1 ;DOI
IDX3   = 0
if (DRUG.EQ.3) IDX3 = 1 ;LSD
IDX4   = 0
if (DRUG.EQ.4) IDX4 = 1 ;quipazine
IDX5   = 0
if (DRUG.EQ.5) IDX5 = 1 ;TFMPP
;Pathway I
logR1 = THETA(5)*IDX1+THETA(6)*IDX2+THETA(7)*IDX3+THETA(8)*IDX4+THETA(9)*IDX5
DDOI  = THETA(6)-THETA(5) ;logRDOI-logRBUF
DLSD  = THETA(7)-THETA(5) ;logRLSD-logRBUF
DQUI  = THETA(8)-THETA(5) ;logRQUI-logRBUF
DTFM  = THETA(9)-THETA(5) ;logRTFM-logRBUF
;Pathway II
logR2 = THETA(10)+(DDOI-THETA(11))*IDX2+(DLSD-THETA(12))*IDX3+(DQUI-THETA(13))*IDX4+(DTFM-THETA(14))*IDX5
;Equilibrium dissociation constant
if (TEST.EQ.1) LKABUF = THETA(15)
if (TEST.EQ.0) LKABUF = 0         ;indicate the pesudo full agonism in IP
if (TEST.EQ.1) LKATFM = 0         ;indicate the pesudo full agonism in AA
if (TEST.EQ.0) LKATFM = THETA(19)
logKA = LKABUF*IDX1+THETA(16)*IDX2+THETA(17)*IDX3+THETA(18)*IDX4+LKATFM*IDX5

n1 = THETA(20) ; hill slope for IP
n2 = THETA(21) ; hill slope for AA

;Model part
A = 10**LOGA ;conc of agonist
```

```
KA= 10**(logKA)

R1= 10**(logR1)

R2= 10**(logR2)

tau1=R1*KA

tau2=R2*KA

IPRED1 = BASAL1+(EM1-BASAL1)*(A*tau1)**n1/((A+KA)**n1+(A*tau1)**n1)

IPRED2 = BASAL2+(EM2-BASAL2)*(A*tau2)**n2/((A+KA)**n2+(A*tau2)**n2)

;pathway 1 IP:TEST=1

;pathway 2 AA:TEST=0

IPRED = IPRED1*TEST+IPRED2*(1-TEST)

Y=IPRED*(1+ETA(1)*TEST+ETA(2)*(1-TEST))+ETA(3)*TEST+ETA(4)*(1-TEST)

$THETA

…

$OMEGA

…

$EST MAXEVAL=2000 NOABORT SIG=3 PRINT=5

$COV

; Xpose

$TABLE ID LOGA DV DRUG TEST IPRED CWRES ONEHEADER NOPRINT FILE=sdtab40
```

4. Model Evaluation of Example II

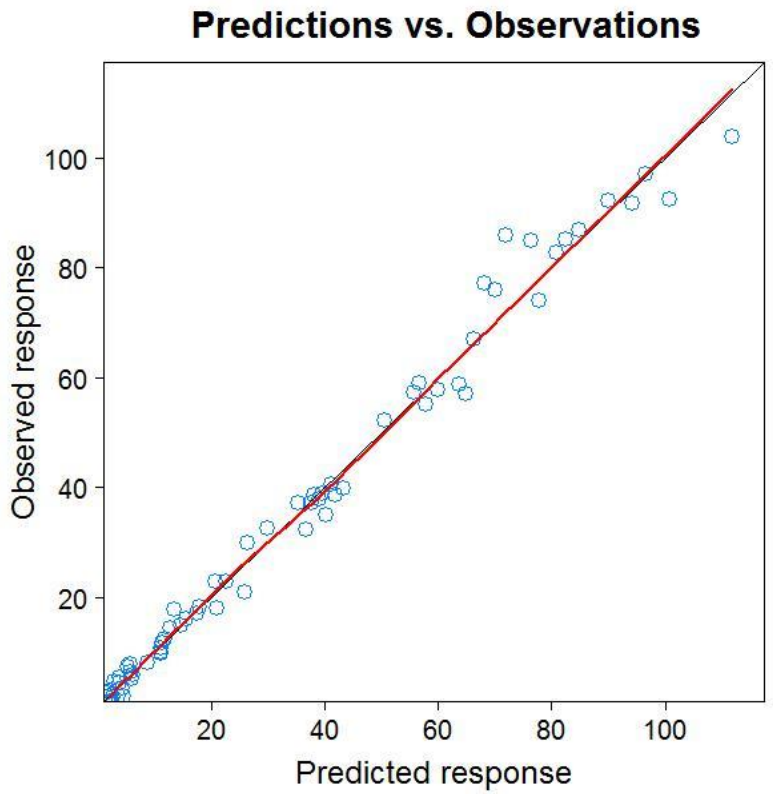

Figure S3. Observed responses vs. predicted responses plot from the intact operational model for Example II.

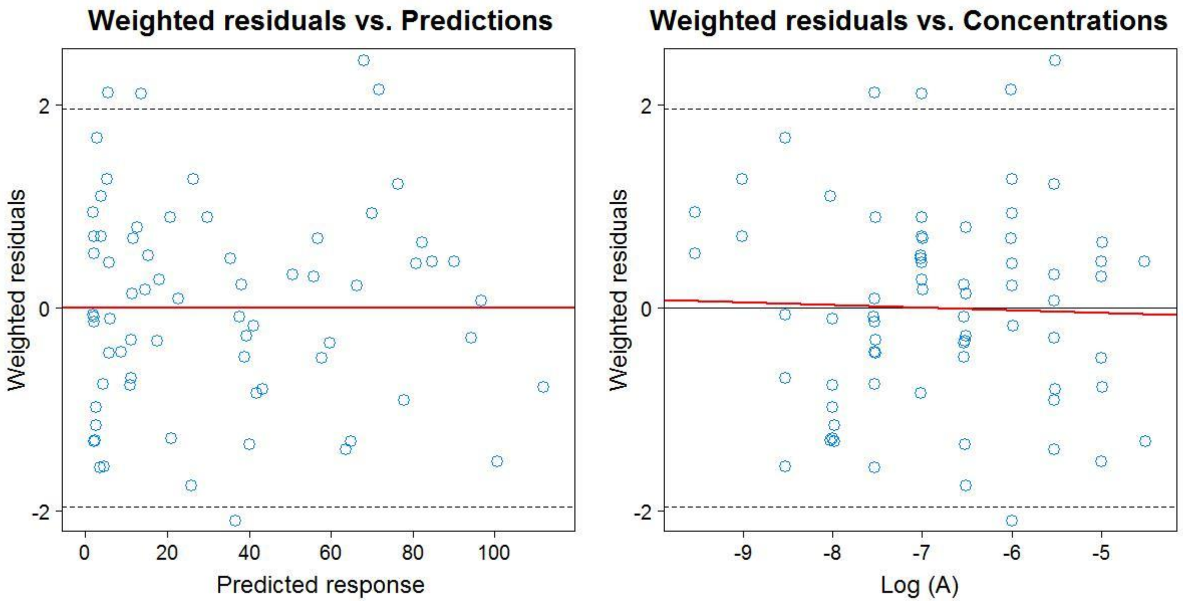

Figure S4. Weighted residuals plots from the intact operational model for Example II. Left: weighted residuals vs. predicted responses. Right: weighted residuals vs. ligand concentrations.

5. *The correlation of C_{50} values between different signalling pathways*

It is noted that the potency of a ligand in different signalling pathways could be highly correlated (Figure S5, data for plotting included in Table S1).

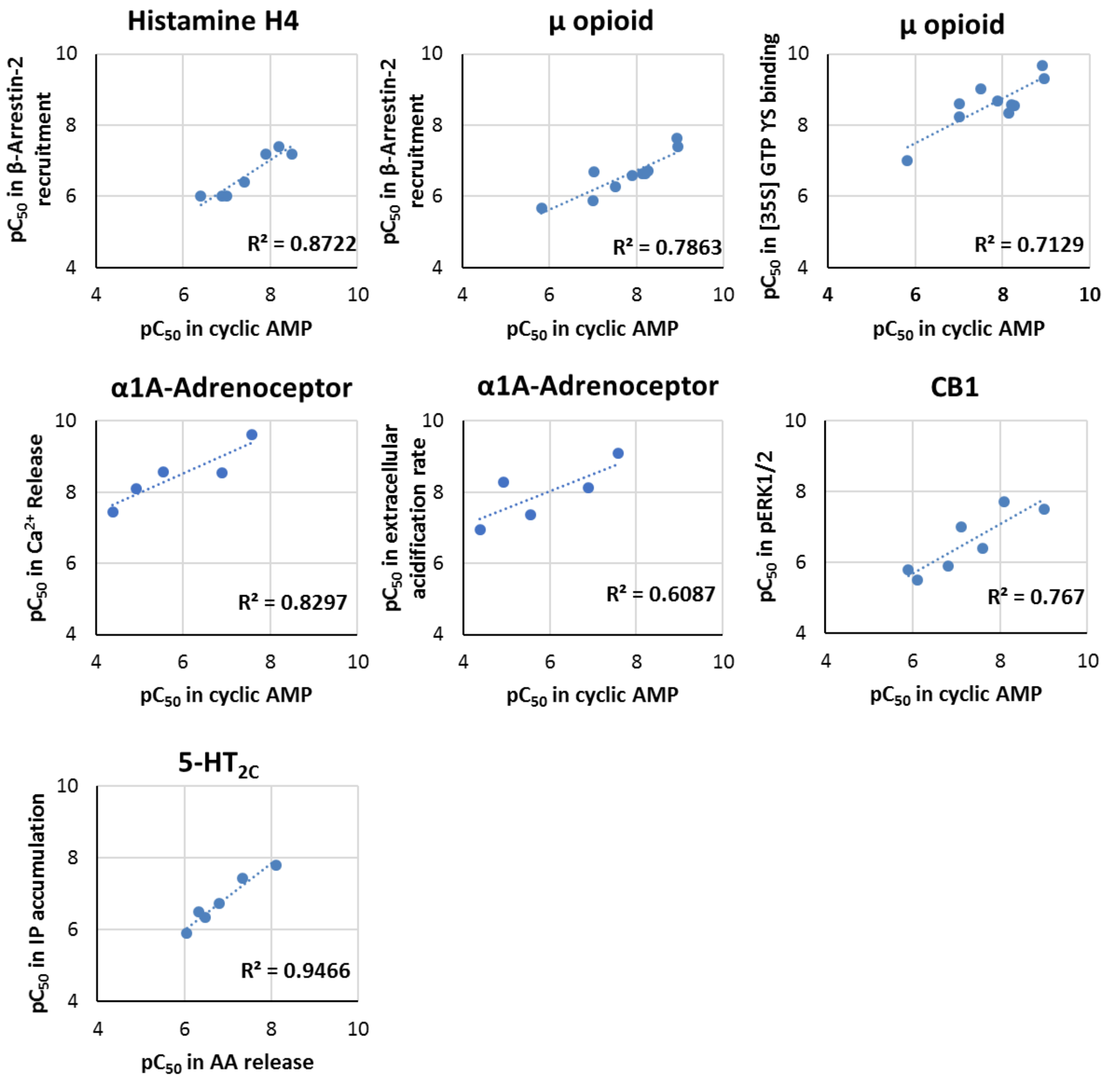

Figure S5. The correlation of pC50 values between different signalling pathways according to GPCR receptors. The data for plotting was detailed in Table S1.

Table S1. The correlation of pC50 values between different signalling pathways according to GPCR receptors

Receptor	Ligand	pC50		r^2
		cyclic AMP	β-Arrestin-2 recruitment	
Histamine H4 (Nijmeijer et al., 2012)	VUF5228	7.40	6.40	0.872
	VUF10185	6.90	6.00	
	VUF10306	6.40	6.00	
	VUF10778	7.90	7.20	
	VUF5222	8.20	7.40	
	VUF4704	7.00	6.00	
	VUF8328	8.49	7.20	
		cyclic AMP	β-Arrestin-2 recruitment	
μ opioid (Winpenny et al., 2016)	Pfizer standard 1	8.92	7.64	0.786
	DAMGO	7.89	6.58	
	Morphine	7.02	6.7	
	Fentanyl	8.14	6.63	
	Oxycodone	5.82	5.68	
	Loperamide	8.95	7.4	
	Endomorphin1	8.26	6.73	
	Endomorphin2	8.2	6.64	
	Met-Enkephalin	7.51	6.28	
	DADLE	7	5.88	
		cyclic AMP	[35S] GTP ϒS binding	
	Pfizer standard 1	8.92	9.67	0.713
	DAMGO	7.89	8.69	
	Morphine	7.02	8.25	
	Fentanyl	8.14	8.34	
	Oxycodone	5.82	7.01	
	Loperamide	8.95	9.31	
	Endomorphin1	8.26	8.56	
	Endomorphin2	8.2	8.57	
	Met-Enkephalin	7.51	9.02	
	DADLE	7	8.61	
		cyclic AMP	Ca^{2+} Release	
α1A-Adrenoceptor (Evans et al., 2011)	Norepinephrine	5.55	8.58	0.830
	Methoxamine	4.39	7.44	
	Phenylephrine	4.93	8.1	
	Cirazoline	6.89	8.54	
	A61603	7.59	9.63	
		cyclic AMP	Extracellular acidification rate	
	Norepinephrine	5.55	7.36	0.609
	Methoxamine	4.39	6.95	
	Phenylephrine	4.93	8.29	
	Cirazoline	6.89	8.13	

	A61603	7.59	9.09	
		**cyclic AMP**	**pERK1/2**	
**CB1 receptor (Khajehali et al., 2015)**	CP55940	8.1	7.7	0.767
	HU-210	9	7.5	
	WIN55,212-2	7.1	7	
	D9-THC	7.6	6.4	
	Methanandamide	6.8	5.9	
	Anandamide	6.1	5.5	
	2-AG	5.9	5.8	
		**AA release**	**IP accumulation**	
**5-HT$_{2C}$ (Berg et al., 1998)**	5-HT	7.34	7.43	0.947
	Bufotenin	6.33	6.5	
	(±)-DOI	6.8	6.72	
	d-LSD	8.11	7.8	
	Quipazine	6.06	5.89	
	TFMPP	6.48	6.34	

References:

Berg KA, Maayani S, Goldfarb J, Scaramellini C, Leff P, Clarke WP (1998). Effector pathway-dependent relative efficacy at serotonin type 2A and 2C receptors: Evidence for agonist-directed trafficking of receptor stimulus. *Mol Pharmacol* **54:** 94-104.

Evans BA, Broxton N, Merlin J, Sato M, Hutchinson DS, Christopoulos A*, et al.* (2011). Quantification of functional selectivity at the human α1A-adrenoceptor. *Mol Pharmacol* **79:** 298-307.

Khajehali E, Malone DT, Glass M, Sexton PM, Christopoulos A, Leach K (2015). Biased Agonism and Biased Allosteric Modulation at the CB1 Cannabinoid Receptor. *Mol Pharmacol* **88:** 368-379.

Nijmeijer S, Vischer HF, Rosethorne EM, Charlton SJ, Leurs R (2012). Analysis of multiple histamine H4 receptor compound classes uncovers Gαi protein-and β-arrestin2-biased ligands. *Mol Pharmacol* **82:** 1174-1182.

Winpenny D, Clark M, Cawkill D (2016). Biased ligand quantification in drug discovery: from theory to high throughput screening to identify new biased mu opioid receptor agonists. *Br J Pharmacol* **173:** 1393-1403.
